# The phylogenomic position of the smooth lanternshark *Etmopterus pusillus* (Squaliformes: Etmopteridae) inferred from the mitochondrial genome

**DOI:** 10.1080/23802359.2016.1172274

**Published:** 2016-04-19

**Authors:** Hao Chen, Xiao Chen, Xichun Gu, Huantong Wan, Xi Chen, Weiming Ai

**Affiliations:** a Department of Marine Biotechnology, School of Life Science, Wenzhou Medical University, Wenzhou, Zhejiang, P.R. China;; b Pearl River Estuary Chinese White Dolphin National Nature Reserve, Zhuhai, Guangdong, P.R. China

**Keywords:** Etmopteridae, *Etmopterus pusillus*, mitochondrial genome

## Abstract

In this study, the complete mitogenome of the Smooth lanternshark *Etmopterus pusillus* (Squaliformes: Etmopteridae) was firstly determined. It was 16,729 bp, consisting of 13 protein-coding genes (PCGs), 22 tRNA genes, 2 rRNA genes and 1 putative control region with typical order to that of most other vertebrates. Its nucleotide base composition is 31.3% A, 23.0% C, 14.3% G and 31.5% T. Two start codons (GTG and ATG) and two stop codons (TAG and TAA/T) were used in the protein-coding genes. The phylogenetic results showed that *E. pusillus* was clustered to *Squaliolus aliae* (Dalatiidae) and suggested that Dalatiidae was polyphyletic.

As one widely distributed species of shark, the smooth lanternshark *Etmopterus pusillus* (Squaliformes: Etmopteridae) was commonly found on or near the bottom of continental and insular slopes at depths from 275 to 1000 m (possibly to 2000 m) in Western & Eastern Atlantic, Western Indian ocean and Western Pacific (Compagno 1984; Last & Stevens 1994). It was ovoviviparous and distinct pairing with embrace (Breder & Rosen 1966) and fed on fish eggs, lanternfish, squid as well as other small dogfish (Compagno et al. 1989). In this study, we firstly determined the complete mitogenome of *E. pusillus* and analyzed its phylogenetic relationship within the Squaliformes.

One specimen of *E. Pusillus* was captured from continental shelf in East China Sea and landed on a pier in Wenling, Zhejiang, China. It was preserved in the museum of marine biology in Wenzhou Medical University with voucher WL2012051264. The experimental protocol and data analysis methods followed Chen et al. (2014). The outgroup *Chimaera monstrosa* and 14 species of Squalimorphs with complete mitogenomes available in the GenBank were selected to construct the phylogenetic tree by Bayesian method (using three partitions: 12S and 16S rRNA genes, the first and second codons of 12 heavy strand encoded protein genes).

The complete mitogenome of *E. pusillus* (Genbank Accession Number: KU892588) is determined to be 16,729 bp in length, consisting of 13 protein-coding genes, 22 tRNA genes, 2 rRNA genes and 1 putative control region, with an identical gene composition, arrangement and transcriptional orientation as most mitogenomes of vertebrates. Its nucleotide base composition is 31.3% A, 23.0% C, 14.3% G and 31.5% T. Between gene junctions exist 25-bp short intergenic spaces and 29-bp overlaps. Except for the *CO*1 gene that starts with the GTG codon, all remaining protein-coding genes use the typical ATG codon as initial codon. Although, 2 of 13 protein-coding genes (*ND*3 and *ND*5) terminate with the TAG codon, others all terminates with the TAA/T codon. The mitogenome of *E. pusillus* contains 22 typical tRNA genes [ranging from 67 (tRNA-*Cys*, *Ser*2) to 75 (tRNA-*Leu*1)], among which except for the tRNA-*Ser*2, all remaining tRNAs could fold into a typical clover-leaf secondary structure. Both 12S rRNA (955 bp) and 16S rRNA (1670 bp) genes were between tRNA-*Phe* and tRNA-*Leu1* genes, separated by tRNA-*Val* gene. The control region is 1081 bp with a rich A + T content (65.8%) and a poor C content (13.1%).

In the Bayesian tree, all nodes are strongly supported (Figure 1). Four orders and seven families are presented. The main basal division is between Hexanchiformes and remaining orders. Pristiophoriformes has closer relationship with Squaliformes rather than Squatiniformes. Within the order Squaliformes, *Etmopterus pusillus* is placed as sister to *Squaliolus aliae* (Dalatiidae), which belongs to the same family as *Somniosus pacificus* (Dalatiidae) does in the morphological classification. However, *Somniosus pacificus* clusters to the species of Squalidae. It suggests that Dalatiidae is polyphyletic. Therefore, the relationship within the order Squaliformes needs further study with more species.

**Figure 1.**
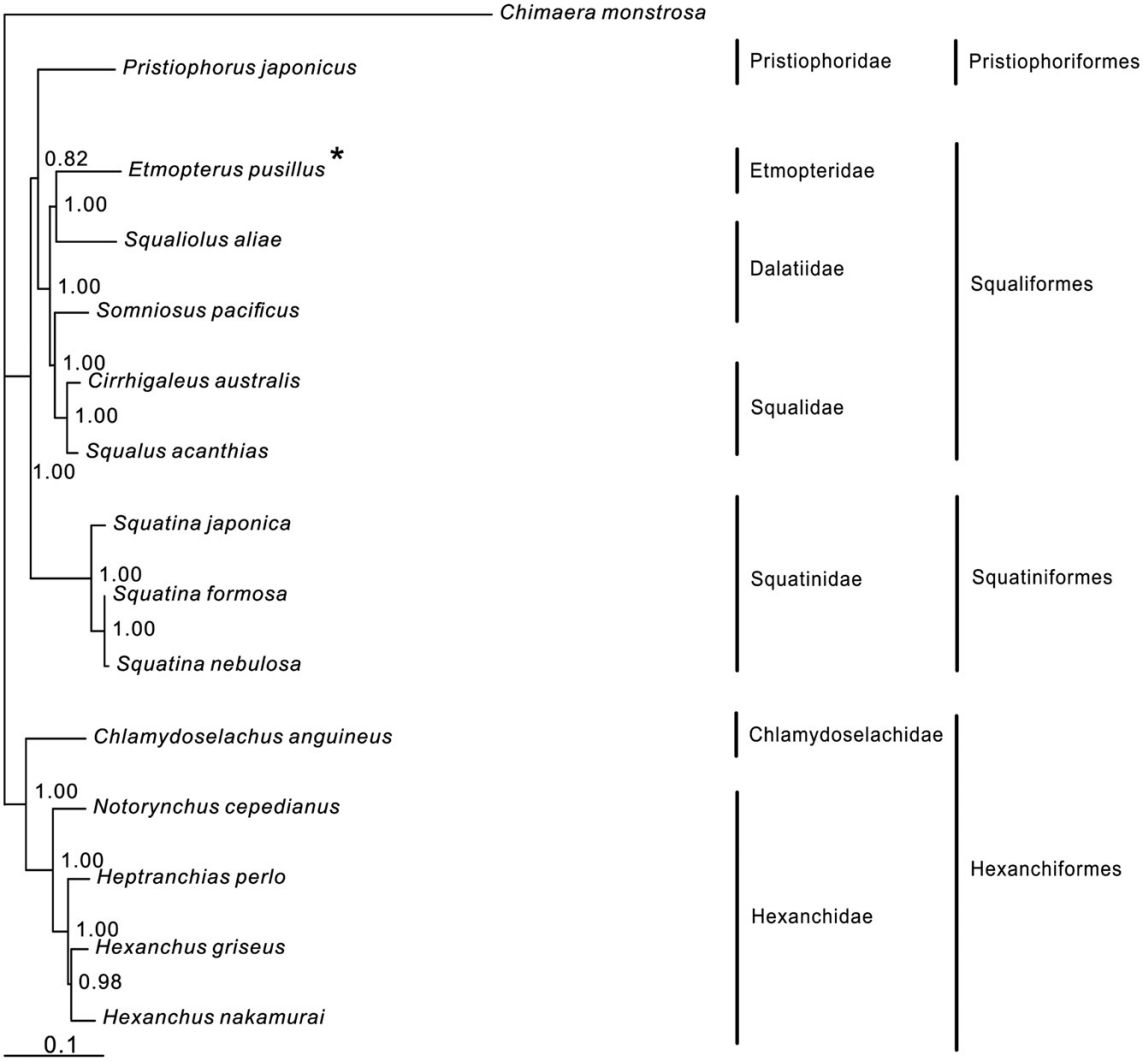
Phylogenetic position of *Etmopterus pusillus Chimaera monstrosa* (AJ310140.1) is selected as the out group. One species from the order Pristiophoriformes is: *Pristiophorus japonicus* (NC_024110.1). Five species from the order Squaliformes are: *Somniosus pacificus* (NC_022734.1), *Squaliolus aliae* (KU873080), *Etmopterus pusillus* (KU892588), *Cirrhigaleus australis* (KJ128289) and *Squalus acanthias* (NC_002012.1). Three species from the order Squatiniformes are: *Squatina japonica* (NC_024276), *S. nebulosa* (NC_025578.1) and *S. formosa* (KM084865). Five species from the order Hexanchiformes are: *Chlamydoselachus anguineus* (NC_022729), *Heptranchias perlo* (NC_022730), *Hexanchus griseus* (KF894491), *H. nakamurai* (AB560491) and *Notorynchus cepedianus* (NC_022731).
